# Perfusion-CT monitoring of cryo-ablated renal cells tumors

**DOI:** 10.1186/1756-9966-28-138

**Published:** 2009-10-10

**Authors:** Ettore Squillaci, Guglielmo Manenti, Carmelo Cicciò, Francesca Nucera, Pierluigi Bove, Giuseppe Vespasiani, Laura Russolillo, Giovanni Simonetti

**Affiliations:** 1 Department of Diagnostic and Molecular Imaging, Interventional Radiology and Radiotherapy - University Tor Vergata, Rome, Italy; 2 Department of Urology, University Tor Vergata, Rome, Italy

## Abstract

**Background:**

No single and thoroughly validated imaging method in monitoring of cryoablated renal cell carcinoma (RCC) is available. The purpose of our study was to determine the feasibility of dynamic contrast-enhanced perfusion CT (pCT) in evaluating the hemodynamic response of RCC.

**Methods:**

15 patients (14 male, 1 female; age range, 43-81 years; mean age, 62 years) with cryoablated RCC via a transperitoneal approach, underwent to pCT 6-8 months after cryo-therapy. pCT was performed for 65 seconds after intravenous injection of contrast medium (80 mL, 370 mg iodine per millilitre, 4 mL/sec). Perfusion parameters (Time/Density curve; Blood flow, BF; Blood Volume, BV; Mean Transit Time, MTT; Permeability-Surface Area Product, PS) were sampled in the cryoablated tumor area and in ipsilateral renal cortex using deconvolution-based method. A tumor was considered to be not responsive to treatment by CT evidence of pathological contrast enhancement in the cryoablated area or renal mass persistence compared with the preoperative CT control. Written informed consent was obtained from all participants before the study.

**Results:**

After cryotherapy, successfully ablated tumor (n = 13) showed decrease in BV (5,39 +/- 1,28 mL/100 g), BF (69,92 +/- 20,12 mL/100 g/min) and PS (16,66 +/- 5,67 mL/100 g/min) value and increased value of MTT (25,35 +/- 4,3 sec) compared with those of normal renal cortex (BV: 117,86 +/- 31,87 mL/100 g/min; BF: 392,39 +/- 117,32 mL/100 g/min; MTT: 18,02 +/- 3,6 sec; PS: 81,68 +/- 22,75 mL/100 g/min). In one patient, assessment of perfusion parameters was not feasible for breathing artifacts. One tumor showed poor response to treatment by the evidence of nodular contrast enhancement in the region encompassing the original lesion. Two typical enhancement patterns were obtained comparing the Time-Density curves of responsive and not responsive ablated tumors.

**Conclusion:**

Perfusion CT seems to be a feasible and promising technique in monitoring the effects of cryoablation therapy.

## Background

Renal cell carcinoma (RCC) is the most common type of kidney cancer (8.100 deaths and 33.130 expected new cases in United States for the 2007), encompassed among highly vascularised tumors [1,2]. Furthermore the common use of cross-sectional imaging method in clinical practise has increased the detection of incidental small RCC [3,4].

Minimally invasive treatments as cryoablation or radioablation have been proposed as a promising alternative to partial or total nephrectomy in selected cases, especially in patients who are poor candidates for conventional surgical resection. Cryoablation of renal tumors can be performed at open, laparoscopic, retroperitoneoscopic surgery and with imaging guided (Computed Tomography, CT; Magnetic Resonance Imaging, MRI) percutaneous approaches. By the evidence of effectiveness in renal tumor constraining of these new thermal therapies, attention is focused to identify a reliable marker of early residual tumor and a feasible imaging monitoring protocol.

Vascularity degree of RCC is known as a prognostic factor correlated with clinical and pathologic stage, metastatic risk and histopathologic grade and it is a significant predictor of disease-specific outcome after therapy [5].

Although a standardized and thoroughly validated method to evaluate tumor vascularity is not available, some biomarkers have been currently proposed as indexes of tumor angiogenic activity. In particular, significant increase of micro vessel density (MVD) and high expression and secretion of vascular endothelial growth factor (VEGF), have been reported in tumor tissue [6]. However, the serial evaluation of these biomarkers as indexes of tumor activity, needs multiple biopsies and is limited because of its invasiveness especially during a long-term follow-up.

An ideal test should be non-invasive, fast, easy to perform, repeatable and reproducible, and most importantly, it should provide in vivo early evidence of residual tumor after therapy and comprehensive data of the tumor structure with informations on tumor angiogenesis functional status.

New imaging modalities (MRI, CT) may be used to obtain informations about microvascular circulation and neoangiogenesis. CT is the imaging technique of reference in surveillance after renal tumor ablation as its ability to distinguish residual tumor (nodular enhancement within the ablated lesion) from successfully cryo-ablated lesion (hypoattenuating areas without focal contrast enhancement with progressive decrease in size).

Therefore, deconvolution-based perfusion computed tomography (pCT) is a non invasive and fast new CT technology that allows measurement of tumor vascular physiology analyzing the time course of tissue enhancement using sequential CT acquisitions during bolus injection of a contrast medium.

This technique generates functional maps and represents in a color scale pixel values the following perfusion parameters: blood flow (BF), blood volume (BV), mean transit time (MTT) and vascular permeability- surface area product (PS). This type of study can also be repeated at different time periods to assess tumor response to thermal therapy to detect temporal changes in tumor angiogenesis.

In our study, we aimed to examine the feasibility of deconvolution-based pCT in monitoring cryoablated RCC and to evaluate whether perfusional CT parameters correlate with response to therapy.

## Methods

### Population

Between May 2007 and June 2008, 15 patients (14 male, 1 female; mean age, 62 years; age range, 43-81 years), underwent to laparoscopic cryoablation for renal tumors (12 renal cell carcinoma, 3 angiomyolipoma), were enrolled in pCT monitoring protocol. In each patient the tumor mean size was 2,04 cm (range 1,5-2,9 cm), showing heterogeneous contrast enhancement in pre-treatment contrast enhanced CT or MRI, not extended beyond Gerota fascia and with no evidence of distant metastases.

The meantime interval between cryoablation procedure and post-therapeutic pCT was 6-8 months. Pre-treatment enhanced CT or MRI images were used as a reference for identification of primitive lesion. Additionally, approximately 6 months postoperatively, CT directed core needle biopsies of the cryoablated tumor were obtained for histophathological examination. All patients were informed of the investigational nature of the study and signed a written consent for participation in accordance with institutional guidelines.

### Cryoablation Procedure

All the patients underwent to laparoscopic cryoablation of the renal lesion via a transperitoneal approach. Briefly, our technique include: an open access through the umbilicus, kidney mobilization, visualization of the entire exophytic aspect of the tumor surface, excision of the overlying fat for pathological examination, imaging of the tumor and entire kidney with a steerable laparoscopic ultrasound (US) probe, guided core needle biopsy of the tumor and, finally, puncture renal cryoablation under laparoscopic and real-time intracorporeal sonographic guidance. According to literature data, our goal was to engulf completely the renal tumor in the iceball further extending the iceball margins approximately 1 cm beyond the tumor edge [7].

Intraoperative pre-cryoablation needle biopsy confirmed renal cell carcinoma (RCC) in 11 patients (73%) and miscellaneous conditions in the remaining 4 patients (27%), including normal kidney tissue in 1, fibrous tissue in 1, angiomyolipoma in 1, oncocytoma in 1.

### Perfusion CT (pCT) technique

Perfusion study was performed with a 64 multi-detector row CT scanner (LightSpeed VCT; GE Medical Systems, Milwaukee, USA). Unenhanced low-dose CT of the upper abdomen (120 kVp, 180 mA, slice thickness 5 mm, 0,6-second gantry rotation time, acquisition mode 27.50/1.375:1, large FOV, matrix 512 × 512) was performed in quite respiration to localize the side of cryoablated tumor. The images were then analyzed by an expert radiologist (ES) experienced in renal tumours, with scans planned to a 40-mm acquisition range for pCT to include the maximum cryoablated area visible. A single radiologist (GM) with a 5 years practice in perfusion CT supervised the acquisition of all the perfusion studies.

The anatomical coverage of pCT is limited on the z-axis, as the acquisition is performed in static table position with a scan range of 40 mm. pCT was performed with cine technique with a delay time of 7 sec after the injection of 80 mL non-ionic iodinated contrast material (iopromide, Ultravist 370; Bayer-Schering), followed by 40 mL of saline solution, injected at a rate of 4 mL/sec by an 18-20 Gauge cannula in the antecubital vein with automatic injector (Stellant, Medrad, Pittsburg, Pa). First-pass scan was obtained with a sampling rate of 1 acquisition per second with a time duration of 45 seconds. After a 25 seconds, a delayed-phase was acquired at the same level with a time duration of 20 seconds. The CT was acquired during quiet respiration and continued for a total time of 65 seconds.

The following parameters were used for dynamic study: eight contiguous 5 mm sections at the same table position, 1-second gantry rotation time, 120 kVp, 80 mA, and 65-seconds acquisition time. The images were reconstructed at a 5 mm thickness and 0,5 sec intervals. The mean effective dose for each patient was about 13 mSv.

### Image Analysis

Data acquired during cine scan were transferred onto an image processing workstation (Advantage Windows 4.4; GE Medical Systems) provided with commercially available software for functional analysis with deconvolution-based technique (Perfusion 3; GE Medical Systems).

The software, after the selection of a threshold value to exclude bone density from the measurements, required to manually or automatically identify arterial input function (AIF) of contrast medium concentration by a 10 mm^2 ^(18-20 pixel area) region of interest (ROI) manually drawn in the abdominal aorta which was always enclosed in the field of view. Selecting a perpendicular-to-section running artery, it was possible to avoid partial volume artifacts that may underestimate reference blood density, leading to misreporting tissue perfusion data.

Then, the software generates Time/Density (Second/Hounsfield Unit) curves from standardized circular regions of interest (ROIs; 10 mm^2^; 18-20 pixel range) manually positioned in the cryoablated area. Care was taken to embed as much of the solid portions of the tumor as possible in order to exclude the necrotic regions and to avoid tumor limits exceeding to exclude peritumoral hyperaemia. Similar circular ROI was placed in healthy omolateral parenchyma as a control to assess perfusion differences between tumor lesion and normal parenchyma.

The graphic representation of contrast perfusion during and immediately after the administration of contrast material was used to obtain the following quantitative parameters of contrast enhancement kinetic:

- Time of arrival (TA), the time between the beginning of dynamic acquisition and onset of enhancement

- Time to peak (TTP), time between onset of enhancement and maximum enhancement

- Peak contrast enhancement (PCE), maximum density value

- Wash-in-rate (WIR), peak value after tissue enhancement - CT value of baseline/time period reaching the tissue peak value from baseline.

Therefore, the software provided in a colour scale pixel, maps of functional parameters for blood flow (BF), blood volume (BV), and mean transit time (MTT) using the central volume principle [8,9]. The capillary permeability-surface area product (PS) was calculated according to the following equation: PS = - blood flow [ln (1- E)], where E is the extraction fraction (the fraction of contrast material that leaks into the extravascular space from the intravascular space) [10].

Contrast-enhanced images were superimposed on the colour map in order to facilitate visual identification of the cryoablated area. BF (in millilitres per 100 g of wet tissue per minute) is defined as the flow rate of blood through the vascular net in a tissue. BV (in millilitres per 100 g of wet tissue) is the volume of blood within the vascular net of a tissue that was flowing and not stagnant. Mean transit time (in seconds) corresponds to the average time taken by the blood elements to traverse the vasculature from the arterial end to the venous end. PS (in millilitres per 100 g of wet tissue per minute) is the product of permeability and the total surface area of capillary endothelium in a unit mass of tissue representing the total diffusion flux across all capillaries.

The pCT is based on a tracer kinetic analysis in which enhancement of the tissue (HU), sampled during arrival of the contrast agent by cine CT scanning, is linearly proportional to the concentration of contrast agent in the tissue. Thus, the time-attenuation curves for the regions of interest were analyzed by means of a mathematical deconvolution method that takes advantage from this linear relationship between the iodine concentration and the CT attenuation numbers.

In particular, deconvolution method uses arterial input function (AIF) to which compare the curve obtained on parenchimal ROIs so as to correct the effect of bolus dispersion and better reflect the tracer kinetic model, which requires an instantaneous bolus input and tissue time-attenuation curves to calculate the impulse residue function (IRF) which is the time enhancement curve of the tissue due to an idealized instantaneous injection of one unit of tracer. It is characterized by an instantaneous peak to a plateau, as the contrast material enters and remains within the tissue, followed by decays as the contrast material washes out from the tissue. The height of the function gives the tissue blood flow (BF) and the area under the curve determines the relative blood volume (BV) [11-13].

Deconvolution analysis is most widely used in acute cerebrovascular disease in which the blood brain barrier is intact. However, in oncologic disease the neo-angiogenesis induces the formation of immature and leaky vessels, which results in heterogeneous microcirculation with interstitial diffusion of tracer (contrast agent). This requires a correction method, as proposed by Nabavi et al [14], in assessing PS parameter according to the Renkin-Crone equation, E = 1 - exp (-PS/BF), to avoid inaccurate determination of blood flow when compartment model is used.

According to a previous study [15], tumor was considered successfully ablated by no evidence of enhanced focal masses within the treated lesion that frequently decreases in size. Perfusion parameters were obtained in tumor cryoablated area and in normal ipsilateral renal cortex to verify the changes in perfusion parameters due to cryo-therapy. No post-procedural biopsy was performed on any tumor. Hence a small number of patients were enclosed in our preliminary study, no statistical analysis was performed.

## Results

**Figure 1:**
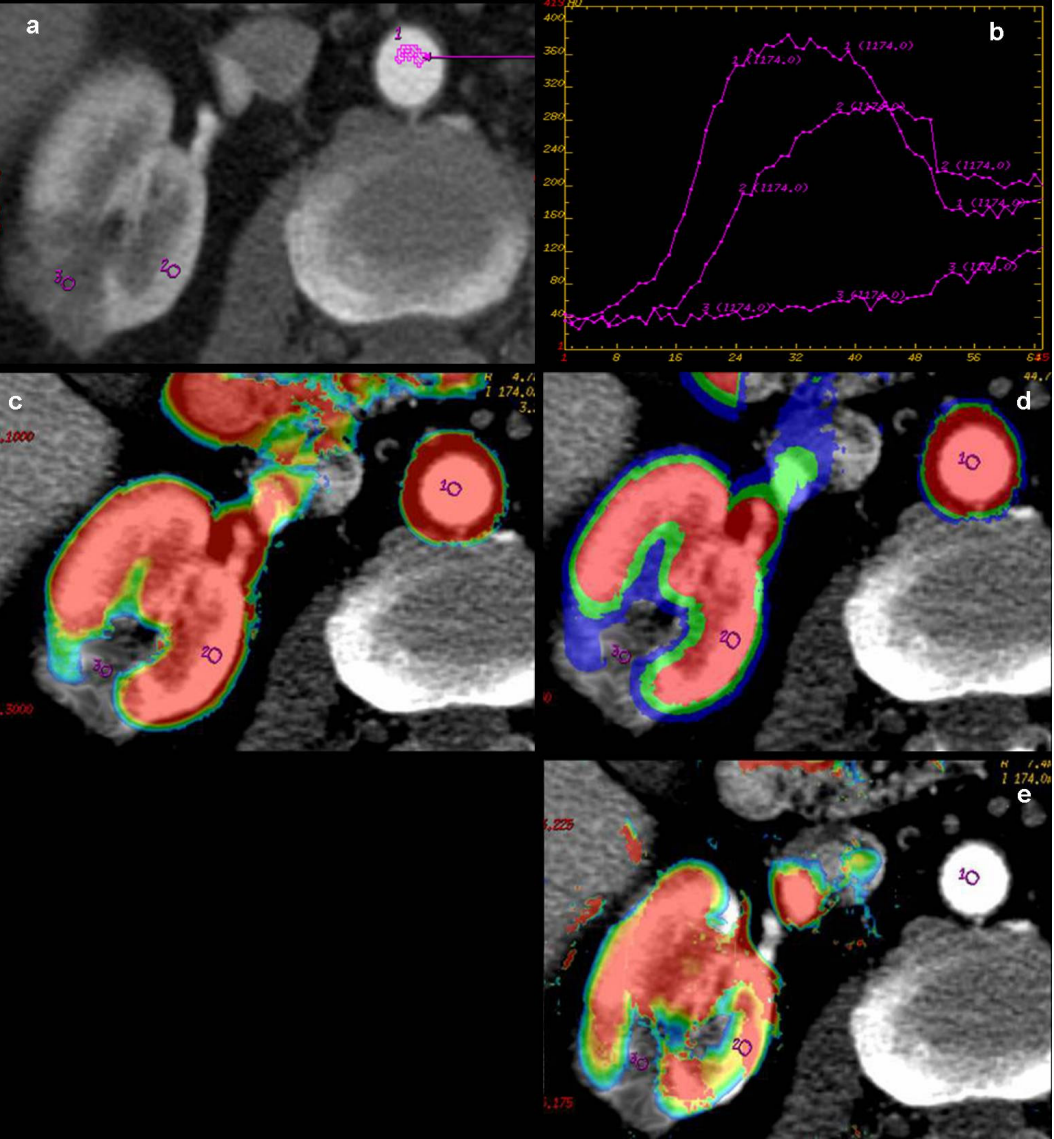
**Cryoablated Renal Cell Carcinoma (RCC) in the right kidney of a 47 years-old patient**. a) Perfusional CT scan shows three regions of interest, selected on abdominal aorta (ROI 1), normal ipsilateral renal cortex (ROI 2), cryoablated tumor area (ROI 3). b) The corresponding time-density curves show contrast enhancement kinetic with typical pattern at responsive cryoablated tumor area (curve 3: slower initial enhancement, decreased amplitude, slower wash-out) compared to abdominal aorta (curve 1) and ipsilateral normal renal cortex (curve 2). Blood colour maps (c, Blood Volume, BV; d, Blood Flow, BF; e, Permeability - Surface Area Product, PS) at the same levels, show the high arterial (ROI 1) and parenchymal (ROI 2) perfusion parameters with no colour encoding in successfully cryoablated area (ROI 3).

**Figure 2:**
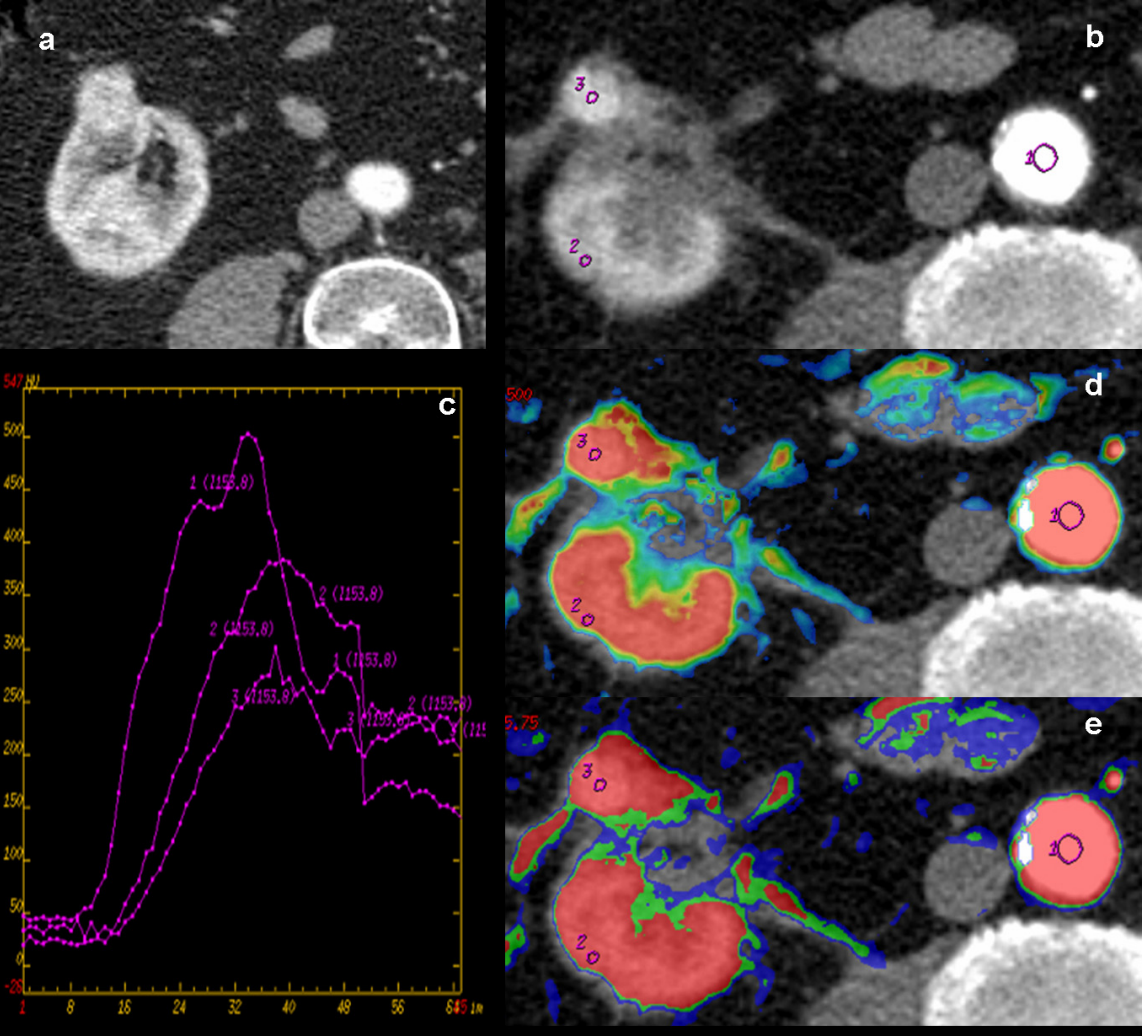
**Residual renal cancer cell (RCC) in right kidney, six months after cryoablation**. Pre-treatment contrast-enhanced cortico-medullary phase CT scan (a) shows exophytic solid tumor with heterogeneous contrast-enhancement. Post-treatment perfusional CT (b) shows a nodular enhancing component (ROI 3) in the medial portion of the ablation zone with peripheral linear enhancement in the peri-renal fat, suggestive for residual tumour. Regions of interest were placed in abdominal aorta (ROI 1), normal renal cortex (ROI 2) and nodular solid enhancement area (ROI 3) to measure perfusional parameters. Time/Density curve (c) shows a typical contrast enhancement pattern in residual tumour area with fast and early wash-in, a plateau trend and a slow, progressive and uniform wash-out (curve 3). Color maps superimposed on gray-scale images (d, Blood Volume, BV; e, Blood Flow, BF) of right kidney show high colour encoding in corresponding residual tumour area: (d) BV (mean, 140,68 ± 24,48 mL/100 g wet tissue/min), (e) BF (mean, 562,72 ± 97,96 mL/100 g wet tissue).

Good image quality was obtained in 14 of 15 patients. 1 Patient had technically inadequate pCT examination due to motion artifacts with data not included in the analysis. 1 patient showed residual tumour. The perfusion parameters (TA, TTP, wash-in rate, Peak contrast enhancement and BV, BF, PS and MTT) in the cryoablated area and normal renal parenchyma of 14 patients were calculated and comparatively evaluated (Table 1, 2). Two pattern curves with different morphology were generated analyzing Time/Density plots. A particular pattern (Type 1), characterised by rapid density increase and tendency to decrease after density peak, was observed in the patient (n = 1) with evidence of residual tumor (Figure 1). A second characteristic curve (Type 2), with steady density increase or a plateau following an initial rise, was identified in patients (n = 13) responsive to treatment, with no evidence of residual tumor (Figure 2).

**Table 1:** Quantitative parameters of contrast enhancement kinetic between responsive cryoablated area and local tissue recurrence.

**Parameters**	Tumor recurrence* [normal omolateral cortex]	Cryoablated area* [normal omolateral cortex]
Time of arrival, TA (s)		
	14,3	15,96 ± 1,29
	[13,8]	[14,85 ± 0,65]

Time to peak, TTP (s)		
	38,3	59,13 ± 2,87
	[39]	

Wash-in rate (1/s)		
	11,52	0,66 ± 0,41
	[9,41]	[7,04 ± 1,35]

Peak contrast enhancement (HU)		
	300,3	60,91 ± 14,85
	[374,18]	[281,77 ± 37,6]

*Values are expressed as mean ± standard deviation (SD).

**Table 2:** Perfusion parameters in recurrent tumor and successfully cryoablated area compared to normal ipsilateral renal cortex value (in square brackets).

**Parameters**	**Recurrent tumor** [normal omolateral cortex]*	**Cryoablated area** [normal omolateral cortex]*
Blood Volume (BV; mL/100 g wet tissue)		
	140,68 ± 24,48	5,39 ± 1,28
	[116,14 ± 14,27]	[117,86 ± 12,53]

Blood Flow (BF; mL/100 g wet tissue/min)		
	562,72 ± 97,96	69,92 ± 20,12
	[393,8 ± 59,01]	[392,28 ± 117,32]

Permeability- Surface Area Product (PS; mL/100 g wet tissue/min)		
	73,52 ± 28,1	16,66 ± 5,67
	[41,88 ± 19,89]	[81,68 ± 22,75]

Mean Transit Time (MTT; sec)		
	15 ± 0,1	25,35 ± 4,3
	[17,69 ± 0,4]	[18,02 ± 3,6]

*Values are expressed as mean ± standard deviation (SD).

Ablation responders (n = 13) showed a peak contrast enhancement (PCE; HU) in cryoablated area after medium contrast administration with a mean-value of 60,91 ± 14,85 [vs. 281,77 ± 37,6 in ipsilateral normal renal cortex]. In the same group the evaluation of kinetic parameters [vs. ipsilateral renal cortex] showed a time of arrival (TA; sec) of 15,96 ± 1,2 [14,85 ± 0,65], a time to peak (TTP; sec) of 59,13 ± 2,87 [49,4 ± 4,4], a wash-in-rate (WIR; 1/s) of 0,66 ± 0,41 [7,04 ± 1,35] (Table 1). Furthermore in the same cases, a variable trend of reduction in BF, BV, and PS values and increase in MTT values were observed in tumor ablated area compared to normal renal cortex (Table 2). In particular the BV, BF and PS mean values sampled in the cryoablated area were lower than in normal renal cortex (respectively: 5,39 ± 1,28 mL/100 g vs 117,86 ± 12,53 mL/100 g; 69,92 ± 20,12 mL/100 g/min vs 392,28 ± 117,32 mL/100 g/min; 16,66 ± 5,67 mL/100 g/min vs 81,68 ± 22,75 mL/100 g/min). MTT was higher in cryoablated area than normal parenchyma (25,35 ± 4,3 sec vs 18,02 ± 3,6 sec).

On the contrary 1 patient had local residual tumor evidenced by renal mass persistence and pathological contrast enhancement with nodular feature in the cryoablated area (TA 14,3 sec; TTP 38,3 sec; WIR 11,56/sec; PCE 301,23 HU) compared to normal ipsilateral cortex (TA 13,8 sec; TTP 44,4 sec; WIR 9,41; PCE 374,18 HU). The mean BV value at the same residual tumour area was 140,68 ± 24,48 mL/100 g (vs. BV of 116,14 ± 14,27 in normal parenchyma), BF and PS mean values respectively were 562,72 ± 97,96 mL/100 g/min (vs. 393,8 ± 59,01 mL/100 g/min in normal parenchyma) and 73,52 ± 28,1 mL/100 g/min (vs. 41,88 ± 19,89 mL/100 g/min in normal parenchyma). MTT was 15 ± 0,1 sec (vs. 17,69 ± 0,4 sec in normal parenchyma).

At a six months postoperative follow-up, 11 patients (73%) underwent CT guided percutaneous core needle biopsy. Two/Three needle cores were obtained per patient with a spring loaded, 18 gauge core biopsy device. According to pCT results with one case of persistent disease, of 25 needle cores obtained, two specimen of RCC were identified in 1 patients. This patient was scheduled for salvage laparoscopic cryoablation and is currently under image monitoring without actual evidence of local residual or metastatic disease at the 12 months follow-up. In the remaining 23 needle cores available, a varying evidence of irreversible cell death was depicted including: hemosiderin deposits in 10 (43%), coagulative necrosis in 8 (35%), and fibrosis in 5 (22%) cores.

## Discussion

Perfusion imaging is a non-invasive functional technique firstly introduced by Miles [16,17] and implemented for the evaluation of neoplastic disease on account of its diagnostic and prognostic value as observed for treatment response of lymphoma [18] and head-and-neck cancer [19], for predictive malignancy value in pulmonary solitary nodule [20], for monitoring of hemodynamic changes after anti-angiogenic therapy [21]. The growing availability of new multislice computed tomographies (MSCTs) and software programs for post-processing perfusion measurements have allowed additional functional informations regarding flow quantification of cross section areas. As far as we know, there are no published reports about the use of pCT in monitoring of cryoablated RCC.

Cryoablation technique is a thermal minimally invasive treatment, developed as an alternative to conventional surgical resection in patients with selected case of RCC, especially for whom the risk of surgery is too great [9,22-28].

The area of necrosis resulting from cryoablation is directed by cytotoxic effect from intracellular ice crystallization during the active freezing cycles and micro-occlusive tissue ischemia by the active or passive thaw cycle [29]. With time fibrosis occurs and the ablated area decreases in size. Although cryoablation of select renal masses is an effective technique in local tumor control [28,30], the ablated renal tumor area is not excised. Indeed accurate monitoring of the ablation zone and early post-ablative detection of residual or recurrent tumor has a pivotal importance.

CT is a widespread technique in cryoablated renal tumors monitoring allowing morphologic imaging of the kidney during several enhancement phases, in a tri-phases acquisition. The multiphasic acquisition with the new MSCTs provides a representation of each component of contrast enhancement (intravascular and extravascular). Therefore, the use of functional imaging techniques to assess tissue perfusion and permeability allows a more deeply angiogenesis process analysis of the tumor with functional informations that cannot be appreciated from qualitative or quantitative (UH) analysis of static tri-phase contrast enhanced images. Furthermore it implies a margining of factors other than angiogenesis that may influence the quantification of contrast enhancement (e.g. amount of contrast agent, patient weight, cardiac output) [31].

The advent of multislice CT scanner with new perfusion software programs creates a unique opportunity for imaging as a reproducible method to assess, in vivo and more deeply than the qualitative evaluation of contrast enhancement, tumor vascularity for monitoring and possibly predicting clinical response to cryotherapy. Otherwise, the common imaging criteria of lesion shrinkage to assess tumor response to cryotherapy may not be the ideal technique of detecting in vivo activity and clinical outcome of ablation and may be implemented with functional imaging parameters from tumor ablated area to obtain much reliable post-treatment informations.

RCC is a highly vascularised tumor with verified correlation between contrast enhancement measures and microvessel density [32] and between its quantification and prognostic information in early-stage of RCC [15,19].

It is well known that neoangiogenesis is a crucial factor for tumor cell growth and metastatic potential in cancer disease, inversely related with patient survival [33]. This process is characterized by increased microvessel density and microanatomical changes of new vessels related to fenestration of the basement membrane resulting in anomalous tissue perfusion compared to normal parenchyma and an increase in the permeability to large molecules in blood.

Considering that tumor neoangiogenesis induces pathophysiological abnormalities to the hemodynamic environment surrounding the tumor, anomalous tissue perfusion can be qualitatively and quantitative expressed in time-enhancement curves and in colour map measurements in a perfusional contrast-enhanced study [31,34].

The degree of contrast enhancement closely matches with the physiological changes of tumor angiogenesis process such as microvessel density, intratumoral/intravascular volume fraction, the tumor blood flow, the vascular permeability-surface area product and the accessible extravascular-extracellular space, all of which result in increased contrast enhancement related to the intensity of neovascularisation [11,31].

Tumor response to non surgical therapies is closely related to tissue perfusion and local oxygen delivery after treatment, attributed in large part to neoangiogenesis [19,35]. On the contrary, cryoablation destroys tissue, indirectly erasing tumor perfusion by means of microvascular damage-induced ischemia, but to date this has not been demonstrated using pCT. Although actually no single test has been validated for neoangiogenesis measurements, in a previous study perfusion-CT positively related with tumor MVD in neo-vascularised areas of RCC [36].

In the tumor response assessment, common imaging features, used to define successfully cryoablated tumors, relies on shrinkage and no focal contrast enhancement in the treated area at morphology evaluation [15,30,37].

Therefore, some Authors reported a threshold of enhancement (10 HU) to distinguish suspected residual tumor (>10 HU) from successfully ablated zone (<10 HU), mostly after radio-frequency ablation rather than cryoablation [38-41]. This quantitative parameter of favourable imaging outcome has not been confirmed by pathology and only a few studies investigated cryoablated areas specimens during follow-up. Weight J.C. et al [42], provide the largest available series regarding the correlation between imaging findings and pathology results after renal tumors cryoablation with favourable agreement between imaging and pathological essays at a 6-months follow-up. Using the morphologic criterion of central nodular enhancement as a predictive feature of positive biopsy in their series, the sensitivity was 77.8% with a 95.1% specificity, 63.4% PPV and 97.7% NPV.

We found two different trend in Time/Density curves of successfully cryoablated area and residual tumour lesion that may be a practical approach during imaging follow-up in early detection of not responsive disease. Overall, in successfully cryoablated area we identified a typical pattern of contrast-enhancement without arterial wash-in and slow wash-in with a plateau trend. Although just observed in one patient, the contrast enhancement curve of the residual tumour area is defined by a fast and early wash-in, a plateau trend and a slow, progressive and uniform wash-out. In line with these findings, our study also provided a positive correlation between kinetics parameters measured Time/Density curves and quantitative measurement of contrast enhancement (BV, BF, MTT, PS).

Successfully cryoablated area demonstrated decreased value of BV, BF and PS and increased value of MTT compared to the normal renal parenchyma.

These two patterns can be useful to distinguish residual tumor from successfully treated area, which enhances and washes-out slowly. Thus, viable tumors tend to have high contrast-enhancement reflected as in colour scale on parametric images, whereas area responsive to treatment show no change in colour. In our experience, the use of pCT as a sensitive technique in the *in-vivo *detection of tumor angiogenesis can improve the identification of residual tumor after therapy from normal tissues or from peri-tumoral area on the basis that angiogenesis, and its physiological imaging findings, can be used as a marker of viable tumor.

Limitations of this study included the availability of matching post-cryoablation imaging results and pathological specimens. We know that core kidney biopsies have a non-diagnostic rate of 20% [43] and a 20% false-negative rate [44] with Weight J.C. reporting an high predictive value of treatment outcome using only imaging findings [42].

In our study, we observed some non-homogeneous densities (inter- or intralesional) of the treated area, resulting in a wide standard deviations of the perfusion parameters. This heterogeneity could be a pitfall related to perfusion values measures of ROIs (variable in size and location) placed on the cryoablated area. We tried to limit the impact of this heterogeneity by sampling the functional parameters using standard sized ROIs drawn at the same level, including only solid area and by excluding necrotic regions.

In our experience pCT provides direct and early evidence of a therapeutic effect by demonstrating changes in the enhancement curves with a slower initial enhancement, decreased amplitude, slower wash-out (Figure 1). In one case, cryotherapy failure in some tumor areas, may be related to the presence of resistant disease subsequently early detected and submitted to additional treatment for control. Furthermore, as a high sensitivity and high specificity method in evaluation of tumor vascularity [11], pCT may be implemented in pre-treatment imaging protocol for clear identification of patients taking an advantage from antiangiogenic therapy [36]. Otherwise, considering the strong colour encoding of the renal parenchyma due to the kidney's high perfusion rate, the implementation of pre-treatment pCT in common imaging protocols may be a useful tool of tumoral vascular structure characterization aimed to tumor area post-treatment follow-up monitoring.

## Conclusion

pCT can detect minimal focal perfusion changes whether the tumor is shrinking or without tumor volume changes, possibly indicating, as in vivo marker of neoangiogenesis, early reversal of tumor responsiveness to cryotherapy by distinguishing cryoablated areas from normal renal adjacent parenchyma.

New imaging CT scanners coming with user-friendly post-processing software will perform integrated and reproducible measurements based not only on tumor morphology but also on tumor function. In particular, the quantitative assessment of perfusional measurements, superimposed to the common used size-based criteria may improve tumor detection and evaluation of therapeutic response. Optimized protocols need to be defined for reducing motion-related artifacts with the minimum-required dose for fairly perfusion measurements. In this study, the promising role of pCT as predictive method for early detection of residual tumor is considered to be preliminary because of the small number of patients. Therefore, additional studies with larger numbers of patients are auspicable to verify these promising results.

## Competing interests

Authors declare that no conflicting or competing interests, of any nature, exist between the Authors of this work and their Academic activity.

## Authors' contributions

ES: supervised the other contributors and critically revised the manuscript. MG: conceived the study and drafted the manuscript. CC: data gathering acquisition analysis and interpretation. FN: data gathering and study coordination. PB: patients collection. GV: oversight of study design, coordination, and writing. LR: retrieved and reviewed the literature. GS: oversight of study design, coordination, and writing. All authors read and approved the final manuscript.
